# Diagnosis of cystic fibrosis: a high heterogeneity of symptoms and genotypes in a Brazil population

**DOI:** 10.1186/s12887-024-04891-z

**Published:** 2024-07-01

**Authors:** Daniela Gois Meneses, Fábia Regina dos Santos, Anne Jardim Botelho, Luciana Mota Bispo, Camilla Guerra Matos, Vynicius Goltran Sobral Propheta, Alexia Ferreira Rodrigues, Géssica Uruga Oliveira, Angela Maria da Silva, Ricardo Queiroz Gurgel

**Affiliations:** 1 Department of Medicine, Federal University of Sergipe, Aracaju, Sergipe Brazil; 2 Health Sciences Graduate Program, Federal University of Sergipe, Aracaju, Sergipe Brazil; 3 Center for Biological and Health Sciences (NPGME), University Hospital, Federal University of Sergipe, Rua Cláudio Batista 505, Palestina, Aracaju, Sergipe CEP 49060-025 Brazil

**Keywords:** Cystic fibrosis, Variants, Phenotypes, CFTR protein, Diagnosis

## Abstract

**Introduction:**

In highly multiracial populations with inadequate newborn screening, knowledge of the various phenotypic presentations of Cystic Fibrosis (CF) can help reach an early diagnosis. This study aims to describe phenotypes and genotypes at the time of CF diagnosis in a state in the Northeast Region of Brazil.

**Methods:**

Retrospective cross-sectional study. Clinical data were extracted from the medical records of CF patients. Clinical, laboratory, and genotypic characteristics were described for patients admitted to a tertiary referral center between 2007 and 2021.

**Results:**

Fifty-eight (58) patients were included in the study, 53.5% of whom were diagnosed through clinical suspicion. The median age at diagnosis was 4.7 months (IQR: 1.5–14.8 months). Five patients had false-negative results in the newborn screening. Faltering growth was the most frequent clinical manifestation. Bronchiectasis and a history of pneumonia predominated in those older than ten, while thinness, underweight, and electrolyte imbalances were more frequent in children under two. Sequencing of the *CFTR* gene identified 27 genotypes, with at least one class I–III variant in all patients, and nine variants that are rare, previously undescribed, or have uncertain significance (619delA, T12991, K162Q, 3195del6, 1678del > T, 124del123bp, 3121–3113 A > T). The most frequent alleles were p.Phe508del, p.Gly542*, p.Arg334Trp, and p.Ser549Arg.

**Conclusions:**

Malnutrition and electrolyte imbalances were the most frequent phenotypes for children < 2 years and were associated with genotypes including 2 class I–III variants. Rare and previously undescribed variants were identified. The p.Gly542*, p.Arg334Trp, and p.Ser549Arg alleles were among the most frequent variants in this population.

## Introduction

Although cystic fibrosis (CF) is a disease with a monogenic cause, significant variability is observed among patients’ phenotypes due to factors such as the variety of variants in the gene encoding the *Cystic Fibrosis Transmembrane Conductance Regulator (CFTR*) protein, the existence of modifier genes, and environmental factors [1]. Using current genotyping techniques, it is increasingly common to detect new variants, some of which still have unknown clinical significance [2].

The Brazilian population is the product of miscegenation between African, European and Native American peoples, which leads to large allelic heterogeneity of the *CFTR* gene in the country. The amount of allelic heterogeneity varies between states according to the colonization profile of each of the country’s regions [3]. With recent advances in CF treatment using new drugs that target specific variants and classes of variants, better knowledge of allele frequencies in the Brazilian population is essential to provide appropriate treatment [4].

This genotypic diversity may contribute to the occurrence of less common variants associated with non-classical phenotypes, and insufficient knowledge of these clinical manifestations may hinder CF diagnosis [5]. Although newborn screening (NBS) for CF has been implemented throughout Brazil, there are regions where a late diagnosis is still common due to false negatives and failures in the screening process [6]. Given these situations, clinical suspicion of CF still contributes to a significant number of diagnoses, and knowledge of the main phenotypic presentations of the disease helps with this surveillance.

This study aimed to describe the phenotype at the time of CF diagnosis and the genotypes of diagnosed patients in the state of Sergipe, in the Northeast Region of Brazil.

## Materials and methods

Retrospective cross-sectional study. Clinical data were obtained from the medical records of patients admitted to a tertiary referral center between 2007 and 2021. Inclusion criteria for patients were sweat chloride ≥ 60 mmol/L in two samples, or the identification of at least two pathogenic genetic variants related to CF. No patient with a confirmed diagnosis of CF was excluded from the sample.

### Variables analyzed

The study analyzed gender, age at diagnosis, clinical manifestations and anthropometric measurements at diagnosis, NBS, initial biochemical and hematological tests, and genotype.

Clinical manifestations were subdivided into: gastrointestinal (diarrhea, vomiting or regurgitation, meconium ileus, intestinal atresia); hepatic (cholestasis, elevated liver enzymes, ultrasound changes); pancreatic (steatorrhea, pancreatitis, diabetes); electrolyte imbalances (hyponatremia, hypochloremia, hypokalemia); pulmonary (chronic cough, recurrent wheezing, cases of pneumonia, bronchiectasis); upper airway disease (recurrent sinusitis, nasal polyposis); azoospermia; faltering growth (> 1SD drop in the weight/age or weight/height index in the last three months for children < 1 year).

The variants were grouped into six classes according to their functional defects [7] and patients were then divided into three groups:

Group 1 (G1): Patients with class I–III variants in both alleles.

Group 2 (G2): Patients with class I–III variants in the first allele and class IV–V variants in the second allele.

Group 3 (G3): Patients with class I–III variants in the first allele and a variant without a defined classification in the literature in the second allele.

### Diagnostic tests and anthropometry

The NBS algorithm for CF used in Brazil is based on the quantification of immunoreactive trypsinogen (IRT), measured in blood collected on filter paper. Using two altered IRTs, a sweat test is performed to confirm the diagnosis if the chloride level is greater than or equal to 60 mmol/L, in two samples [8]. The NBS was implemented in the public health network in Sergipe from 2014, in the period prior to this year only patients from private services were diagnosed by the NBS.

For the anthropometric evaluation, weight and height were measured according to standardized protocols [9] at the first consultation. The anthropometric indices height/age (H/A), weight/age (W/A), and Body Mass Index (BMI)/age (BMI/A) were calculated using the program WHO ANTHRO version 2.0.2. Stunting was identified in children whose H/A z-score (HAZ) was < -2, and severe stunting was identified when HAZ < -3. Underweight and severely underweight children were recognized by W/A z-score (WAZ) values < -2 and < -3, respectively. Thinness and severe thinness were identified when BMI/A z-scores were < -2 and < -3, respectively [10]. Patients over 18 were considered thin if they had BMI values < 18.5 kg/m2 and overweight if their BMI was ≥ 25 kg/m2 [11]. Birth weight was used as the first anthropometric reference to check for faltering growth in children < 1 year [12].

Serum hematological and biochemical evaluations were performed at the diagnosis of CF. Serum electrolyte levels were considered normal within the following ranges: 135–145 mEq/L for sodium, 3–4.5 mEq/L for potassium, and 95–110 mEq/L for chloride [13].

Pulmonary function was evaluated by measuring forced expiratory volume in 1 s (FEV1) and forced vital capacity (FVC), both expressed as % of normal predicted values for the patient’s age, sex, and height. Oropharyngeal secretion samples were collected for microbiological analysis.

### Genetic analysis and classification of variant pathogenicity

DNA samples were extracted from patients’ saliva, and the *CFTR* gene was analyzed by the laboratory Mendelics Análise Genômica S.A. using next-generation sequencing (NGS). The Brazilian Cystic Fibrosis Group provided resources for the gene sequencing. Regions of interest were captured using customized capture probes using Nextera exome capture kits, then sequenced using an Illumina Hiseq platform. Sequence alignment and the identification of variants were performed using the laboratory’s bioinformatics protocols using human genome build GRCh37/UCSC hg19 as a reference.

Pathogenicity was predicted using Mendelics’ proprietary software Abracadabra. Variants identified as pathogenic were compared to their frequencies in control populations using the population banks gnomAD v2 and v3. The pathogenicity of variants not included in the CFTR2 database was classified based on the recommendations of the American College of Medical Genetics (ACMG) [14], and nomenclature standardization followed the recommendations of the HGVS (Human Genome Variation Society).

### Statistical analysis

The hypothesis of independence between categorical variables was tested using Pearson’s chi-squared test. The hypothesis of adherence of continuous variables to a normal distribution was tested with the Shapiro-Wilk test. As this hypothesis was not confirmed, the hypothesis of equality of medians was tested using the Kruskal-Wallis test. Dunn’s test for multiple comparisons was applied to test hypotheses of pairwise equality of medians. All statistical analyses used a significance level of 5% and were performed in R software version 4.2.0, 2022.

### Ethical aspects

The Federal University of Sergipe’s Research Ethics Committee approved this study under CAEE number 40557520.9.0000.5546 and approval # 4.544.859. All patients ≥ 18 years of age and the parents/legal guardians of minor patients signed Free and Informed Consent Forms before participating in the study.

## Results

Fifty-eight patients were included in the study, 30 (51.7%) of whom were females. Forty-four (75.9%) patients were diagnosed at ages < 2 years and 3 (5,2%) with ages > 18 years old, the median age at diagnosis of 0.39 years (IQR: 0.13–1.23). Fifty-five patients (94.8%) had sweat chloride ≥ 60mmol/L, 2 patients had the genotype diagnosis performed by the private network after 2 altered IRTs, 1 patient had sweat chloride with a result between 30 and 60 mmol/L and the diagnosis confirmed by the genotype. One (1.7%) patient was diagnosed by NBS, had 2 sweat chlorides ≥ 60mmol/L, but only 1 pathogenic variant was identified. The remaining patients had the 2 variants identified by NGS.

Thirty-one patients (53.4%) were diagnosed with clinical suspicion, although 9 of them had undergone NBS. The NBS was performed in 36 patients (62%), ten (17.2%) were asymptomatic, five (13.9%) had normal levels of IRT in the NBS and 4 (11.1%) did not have the NBS completed by the private health network. The clinical characteristics and genotype of patients with false negative NBS results are described in Table 1.

**Table 1:** Clinical characteristics and genotype of patients with false negative NBS results

Patients	1	2	3	4	5
Sex	Feminino	Female	Male	Female	Male
Allele 1	p.Phe508del	p.Phe508del	p.Phe508del	p.Gly542*	p.Ser466*
Allele 2	p.Arg334Trp	p.Gly542*	p.val1022_Ile1023del	p.Arg117His	Alelo 5T
IRT (mg/dL)	61,6	55,8	69,4	40,3	33,3
Sweat test (mmol/L)	165/113	107	86/104	38/44	72/90
Age at diagnosis	6 months	4 moths	4 moths	4 years	3 years
Nutritional status at diagnosis	severe thinness, Stunting	severe thinness Adequate stature	severe thinness, Stunting	Thinness Adequate stature	Eutrophy Adequate stature
Meconium ileus	No	Yes	No	No	No
Respiratory symptoms	No	Yes	Yes	No	Yes
Exocrine pacreatic insufficiency	Yes	Yes	Yes	Yes	Yes
Initial symptoms	Faltering growth, Vomiting, diarrhoea, steatorrhea, electrolyte disturbance, anaemia	Faltering growth, diarrhoea, steatorrhea, hepatites, electrolyte disturbance, anaemia	Faltering growth, steatorrhea, electrolyte disturbance.	Vomiting, abdominal pain, pancreatitis	Constipation, hepatitis, pneumonias, anaemia
Consanguinity	No	No	No	No	No
Family history	No	Yes	No	No	No

All patients’ genotypes had at least one variant of classes I, II, or III. These were associated with a second class I–III variant in 35 (60.3%) patients and a Class IV–V variant in 14 (24.1%) patients; in nine (15.5%) patients, the second variant had no classification defined in the literature.

Faltering growth occurred in 36 (62%) patients < 1 year of age and was the most frequent clinical manifestation, followed by pulmonary manifestations in 29 (50%) patients, and gastrointestinal manifestations in 26 (44.8%) patients, most of which presented nonspecific signs and symptoms. Clinical findings more suggestive of CF, such as meconium ileus requiring surgical intervention, bronchiectasis, nasal polyps, and infertility, were uncommon (1.7%, 13.8%, 3.5%, and 3.4% of all patients, respectively). Bronchiectasis, history of cases of pneumonia, and upper airway manifestations were more frequent in patients older than ten, while thinness, underweight, and electrolyte imbalances (EI) were more frequent in patients aged < 2 years (Table 2).

**Table 2:** Clinical manifestations by age group in CF patients

Clinical manifestations, *n* (%)	Age group			
	< 2 years *N* = 44	2 to 9 years *N* = 7	≥ 10 years *N* = 7	*p*-value^C^
Pulmonary	20 (45.5)	3 (42.9)	6 (85.7)	0.194
Chronic cough	20 (45.5)	3 (42.9)	6 (85.7)	0.194
Recurrent wheezing	12 (27.3)	2 (28.6)	2 (28.6)	1.000
Previous pneumonia	4 (9.1)	3 (42.9)	6 (85.7)	**< 0.001**
Bronchiectasis	0 (0)	2 (28.6)	6 (85.7)	**< 0.001**
Gastrointestinal	20 (45.5)	5 (71.4)	1 (14.3)	0.112
Vomiting/regurgitation	14 (31.8)	2 (28.6)	0 (0)	0.322
Diarrhea	14 (31.8)	3 (42.9)	1 (14.3)	0.510
Abdominal pain	3 (6.8)	3 (42.9)	1 (14.3)	0.065
Constipation	1 (2.3)	1 (14.3)	0 (0)	0.431
Intestinal atresia	1 (2.3)	0 (0)	0 (0)	1.000
Clinical meconium ileus	4 (9.1)	0 (0)	0 (0)	0.571
Meconium ileus requiring surgery	1 (2.3)	0 (0)	0 (0)	1.000
Pancreatic	21 (47.7)	3 (42.9)	2 (28.6)	0.744
Steatorrhea	21 (47.7)	2 (28.6)	2 (28.6)	0.445
Pancreatitis	0 (0)	1 (14.3)	0 (0)	0.248
Diabetes	0 (0)	0 (0)	1 (14.3)	0.248
Electrolyte imbalance	21 (51.2)	0 (0)	0 (0)	**0.003**
Hyponatremia	20 (48.8)	0 (0)	0 (0)	**0.006**
Hypochloremia	11 (28.2)	0 (0)	2 (40)	0.377
Hypokalemia	5 (12.2)	0 (0)	0 (0)	0.455
Hepatic	16 (36.4)	1 (14.3)	2 (28.6)	0.650
Prolonged jaundice	1 (2.3)	0 (0)	0 (0)	1.000
Elevated liver enzymes	15 (34.1)	1 (14.3)	0 (0)	0.123
Ultrasound changes	8 (18.2)	1 (14.3)	2 (28.6)	0.848
Upper airways	3 (6.8)	4 (57.1)	7 (100)	**< 0.001**
Recurrent rhinosinusitis	3 (6.8)	4 (57.1)	7 (100)	**< 0.001**
Nasal polyposis	0 (0)	1 (14.3)	1 (14.3)	0.059
Underweight	26 (59.1)	1 (14.3)	1 (14.3)	**0.014**
Stunting/severe stunting	17 (38.6)	1 (14.3)	1 (14.3)	0.265
Thinness/severe thinness	24 (54.5)	1 (14.3)	1 (14.3)	**0.030**

Legend: n – absolute frequency. % – relative frequency. C – Pearson’s chi-squared test

EIs were present in 48.4% (15/31) of the patients diagnosed through clinical suspicion, 25.9% (7/27) of patients diagnosed by NBS, and 60% (3/5) of patients with false-negative results in NBS. One of the patients presented with Pseudo-Bartter’s syndrome and was diagnosed with CF at nine months through genotyping requested upon suspicion of renal tubulopathy.

*CFTR* gene sequencing led to the identification of 23 variants. The most frequent alleles were p.Phe508del – 58 (50%), p.Gly542* – 15 (12.9%), p.Arg334Trp – eight (6.9%), and p.Ser549Arg – seven (6%). A total of 27 different genotypes were found. The most prevalent genotypes were: homozygous p.Phe508del, found in 18 (31%) patients; p.Phe508del/p.Gly542* and p.Phe508del/p.Arg334Trp, found in six (10.3%) patients each; and p.Phe508del/p.Ser549Arg, p.Phe508del/p.val1022_Ile1023del, p.Gly542*/p.Gly542*, and p.Ser549Arg/p.Lys162Glu found in two (3.5%) patients each. The remaining genotypes (34.5%) were found in a single patient each. The distribution of the variants found is shown in Table S1. The frequency of the main alleles found in Sergipe in other regions of Brazil and worldwide is shown in Table 3.

**Table 3:** Pathogenic variants of the *CFTR* gene in Sergipe, in Brazilian regions and world

VARIANTS	Sergipe *n* = 58	Northeast¹ *n* = 646	Southeast¹ *n* = 2.864	South¹ *n* = 1.384	Brazil¹ *n* = 6208	World² *n* = 142.036
p.Phe508del	50%	57.20%	50.30%	50.40%	45.97%	69.74%
p.Gly542*	13%	5.40%	8.00%	7.90%	6.68%	2.54%
p.Arg334Trp	6.9%	2.30%	2.10%	2.10%	1.88%	0.30%
p.Ser549Arg	6%	0.90%	1.20%	0.10%	0.69%	0.065%
3120 + 1G-> A	2.6%	2.30%	4.00%	1.30%	6.68%	0.35%
3237–26 A-> G	1.7%	1.8%	0.90%	0.10%	0.74%	0.33%

Sources: ¹Silva Filho et al., 2020; ²CFTR2 Apr 2022

Pulmonary, gastrointestinal, and pancreatic manifestations had similar distributions across genotypes. Hepatic manifestations (*p =* 0.005), low sodium levels, hypoalbuminemia, low hemoglobin levels, low weight and short height were more frequent in patients with 2 class I–III variants (G1), as seen in Table 4.

**Table 4:** Anthropometric and laboratory markers according to genotype in CF patients

Anthropometric and laboratory markers Median (IQR)	Variant groups				
	G1	G2	G3	*p*-value^k^	
Weight/Age z-score (WAZ)	-2.4 (-4; -1.5) a	-2.1 (-3.2; -0.9) a, b	-0.7 (-1.1; 0) b	0.003	
Height/Age z-score (HAZ)	-1.8 (-3.5; -1) a	-1.4 (-2; -0.1) b	-0.6 (-0.9; -0.1) b	0.005	
BMI/Age z-score	-2.2 (-2.9; -1.3) a	-1.6 (-2.7; -0.1) a, b	-1.1 (-1.4; 0.1) b	0.063	
Hemoglobin	10.1 (9.5; 11.8)	11.6 (10.7; 12.5)	12.4 (10.9; 13.3)	0.038	
Albumin	3.1 (2.6; 3.8) a	4.1 (3.8; 4.3) b	4 (3.8; 4.2) b	0.001	
INR	1.2 (1; 1.5) a, b	1.1 (1; 1.1) a	1.3 (1.3; 1.4) b	0.012	
Sodium	135 (129; 137.5) a	137.4 (127; 140) a, b	139 (139; 142.6) b	0.005	
Chlorine	102 (94; 105)	98.5 (84.5; 102.5)	107 (104; 108)	0.067	
Potassium	4.6 (4.2; 5) a	4 (2.7; 4.5) b	4.9 (4.2; 5.2) a	0.022	

Legend: n – absolute frequency. IQR – Interquartile range. K – Kruskal-Wallis test. a, b – Groups recognized as distinct by Dunn’s test for multiple comparisons. INR – International Normalized Ratio

Nine (15.5%) of the 58 patients evaluated had variants of uncertain significance, or rare or previously undescribed variants (Table 5). Clinical, laboratory, and imaging test results for these patients are shown in Tables 6 and 7.

**Table 5:** Rare or novel variants and variants of uncertain significance found in the study population

Variant	Nucleotide change (cDNA)	Legacy name	Class	Variant type	ACMG classification^a^	Registration in CFTR2^b^
p.Lys163Arg fs*3	c.487delA	619delA	IA	Frameshift	Pathogenic	Absent
p.Thr1299Ile	c.3896 C > T	T1299l		Missense	Uncertain significance	Absent
p.Lys162Glu	c.484 A > G	K162Q		Missense	Uncertain significance	Absent
p.Val1022_Ile1023del	c.3063_3068delAGTGAT	3195del6		In-frame deletion	Pathogenic	Absent
p.Arg516Leu fs*51	c.1546_1548delinsT	1678del3 > T	IA	Frameshift	Pathogenic	Absent
p.?	c.-9_14del23	124del23bp	IA	Start-loss	Pathogenic	Present
p.?	c.2989–313 A > T	3121–3113 A > T		Splicing	Pathogenic	Absent

a Based on the recommendations of the American College of Medical Genetics (ACMG)

b Available at www.cftr2.org

**Table 6:** Clinical data of patients with rare variants of the *CFTR* gene

	Case 1	Case 2	Case 3	Case 4	Case 5	Case 6	Case 7	Case 8	Case 9
Sex	Male	Male	Female	Female	Male	Female	Female	Male	Female
Allele 1	c.487delA	c.2988 + 1G > A	c.1647T > G	c.1647T > G	c.3063_3068delAGTGAT	c.3063_3068delAGTGAT	c.1546_1548delinsT	c.-9_14del	c.2989–313 A > T
Allele 2	c.487delA	c.3896 C > T	c.484 A > G	c.484 A > G	c.1521_1523delCTT	c.1521_1523delCTT	Unknown	c.2053_2054dupA	c.1521_1523delCTT
Age at diagnosis	8 months	15 years	2 months	2 months	5 months	1 month	29 days	2 months	10 years
Current age	15 years	24 years	7 years	7 years	5 years	3 years	2 years	2 years	19 years
Form of diagnosis	Clinical	Clinical	Newborn screening	Newborn screening	Clinical	Newborn screening	Newborn screening	Newborn screening	Clinical
Consanguinity	Yes	Unknown	No	No	No	No	No	No	No
Family history	No	Unknown	Yes	Yes	No	No	No	No	No
IRT (mg/dL)	Not performed	Not performed	106.7	92.2	69.4	145	152	96.2	Not performed
Sweat test (mmol/L)	163/151	120/111	103/89	99/93	86	91/99	98/102	65/81	88/89
Nutritional status at diagnosis	Malnutrition Severe stunting	Normal weight Adequate height	Normal weight Adequate height	Normal weight Adequate height	Malnutrition Stunting	Normal weight Adequate height	Normal weight Adequate height	Normal weight Adequate height	Normal weight Adequate height
Meconium ileus	No	No	No	No	No	No	No	No	No
*Pseudomonas aeruginosa* First infection	11 months	Never	4 years, 4 months	4 years, 11 months	4 months	1 month	2 years, 2 months	6 months	10 years, 7 months
Steatorrhea	Yes	No	No	No	Yes	Yes	Yes	No	No
Initial symptoms	Diarrhea, vomiting, cough, pneumonia anemia	Nasal polyps and nasal congestion.	Cough	Cough	Cough, wheezing, and difficulty gaining weight	Cough and anemia	Vomiting, diarrhea, and difficulty gaining weight	Asymptomatic	Cough, recurrent sinusitis, pneumonia, difficulty gaining weight

**Table 7:** Complementary exams of patients with rare variants of the *CFTR* gene

	Case 1	Case 2	Case 3	Case 4	Case 5	Case 6	Case 7	Case 8	Case 9
FEV1 – FVC	24–32%	41–83%	83–86%	69–79%	Not performed	Not performed	Not performed	Not performed	41–51%
Fecal elastase (mcg/g)	< 15	> 500	408	120	< 15	< 15	< 15	23	> 500
Abdominal Ultrasound	No alteration	No alteration	No alteration	No alteration	No alteration	No alteration	No alteration	No alteration	No alteration
Ecocardiogram	Mild tricuspid insufficiency (PASP 35mmHg)	Not performed	Not performed	No alterations	No alterations	No alterations	No alterations	No alterations	Mild mitral insufficiency (PASP 24mmHg)
Spermogram	Not performed	Azoospermia	Not applicable	Not applicable	Not applicable	Not applicable	Not applicable	Not applicable	Not applicable
Chest CT scan	Bilaterally diffuse cylindrical and cystic bronchiectasis, large cystic formation in the left upper lobe, bilateral diffuse mucoid impaction	Cylindrical and varicose bronchiectasis, mucoid impaction in the anterior segment of the right upper lobe. Areas with air trapping.	No alterations	No alterations	No alterations	Consolidation with interposed air bronchogram in the middle lobe (active infectious process), without other changes in the lung parenchyma	Not performed. Chest X-ray without alterations, a patient without pulmonary exacerbations.	Bronchial thickening and bilaterally scattered bronchiectasis, predominantly on the right lung	Bilateral cylindrical bronchiectasis, diffuse lung wall thickening, mucoid impaction areas, predominantly in the upper lobes. Budding tree pattern in the lower lobes.

## Discussion

The median age at diagnosis, including patients diagnosed by clinical suspicion and NBS, was 0.39 years (IQR: 0.13–1.23), although NBS was only implemented in the public health network in 2014. Similar results to that found in the South Region of Brazil (0.32 years, IQR 0.11–4.25), where NBS has been performed since 2000–2001, but lower than the median age at diagnosis compared to other regions of the country. The median age at diagnosis in the North and Northeast Regions of Brazil, where NBS was implemented at a similar period to its implementation in Sergipe, was 5.07 years (IQR 1.3–10.2) and 2.89 years (IQR 0.43–8.15), respectively [4]. This shows a high level of clinical suspicion of the disease in the studied population, allowing for early diagnosis in an area where NBS diagnosed only 46.7% of patients, and five children with false-negative results in the NBS received a clinical diagnosis.

The high rate of clinical suspicion for CF in our service probably is due to the sum of factors such as: the operation of the only CF referral center in a teaching hospital, at the tertiary level, which provides training on the diagnosis and treatment of the disease to all pediatric resident physicians in the state; the existence of referral services in paediatric gastroenterology, paediatric pulmonology and a referral ward for the treatment of infants and children with severe malnutrition in the same hospital unit; and the availability of the sweat test for all suspected patients treated at this hospital unit.

Although NBS is already widely implemented, diagnosing CF remains very challenging in some situations. The occurrence of false negatives of NBS, the difficulty of completing the various stages of NBS until the diagnostic conclusion occurs, and the possibility of intermediate results of chloride levels in sweat, 30–59 mmol/L, can greatly delay the diagnosis of FC. The determination of the genotype can solve the cases of patients with doubtful results of chloride in sweat, and in these situations NGS is recommended, but the genotype result may be inconclusive, making it necessary to perform additional tests to evaluate the CFTR function that are performed in few research centers [15]. Clinical suspicion of CF in children < 2 years of age with faltering growth and electrolyte imbalances probably favored an early diagnosis of the disease. Hyponatremia was the most frequent EI. These increased sodium losses in babies may occur due to exposure to the high temperatures common in the Northeast Region of Brazil and the predominant intake of breast milk and milk formulas with inadequate salt content to meet the general nutritional needs of CF patients. A study conducted in Brazil found a 95% incidence of hyponatremia in children evaluated at a mean age of 45 ± 18 months of age [16], and a systematic review [17] found EIs at ages < 2.5 years in 75% of 262 patients—60% of the patients were diagnosed with EIs before CF was identified.The diagnosis of CF should be considered in any child with hypochloremic metabolic alkalosis, even if the NBS result is negative. Knowing the phenotype of variants typical of some regions can help the monitoring of patients with inconclusive diagnosis of NBS and guide the inclusion of specific variants in NBS genetic panels, as occurred in the Tuscany region with the S737F variant often associated with hypochloremic metabolic alkalosis in childhood [18–20].

Chronic bacterial airway infections, mucus obstruction of the airways, and progressive bronchiectasis characterize advanced pulmonary disease in CF and lead to greater morbidity and mortality [21]. There is a broad spectrum of presentations of respiratory disease throughout the first years of life, and findings more specific to CF may not occur at first, delaying diagnostic suspicion. In the population analyzed here, bronchiectasis, history of pneumonia, recurrent sinusitis, and nasal polyps were more frequent in patients > 10 years of age and were unrelated to specific genotypes.

Malnutrition, EIs, hypoalbuminemia and/or anemia were more frequent in individuals with two class I–III variants, probably due to an association with exocrine pancreatic insufficiency [22].

The frequency of the p.Phe508del allele in Sergipe was lower than that observed worldwide and in the northeast of Brazil, while the p.Gly542, p.Arg334Trp, and p.Ser549Arg alleles were more frequent in Sergipe. This pattern is probably related to a mixed ancestry, including Europeans, Africans, and Native Americans [23], except for the p.Ser549R variant, which originated in the Middle East region [24, 25].

Two variants previously not described in the literature were identified: p.Lys163Arg fs*3 (c.487delA), and p.Arg516Leufs*51 (c.1546_1548delinsT). P.Lys163Arg fs*3 (c.487delA) was found in homozygosity in a patient with consanguineous parents. Considering the mutational mechanism, this variant may be included as a class I variant, and it may be classified as pathogenic using the ACMG criteria (PVS1 + PM2 + PP4). On the other hand, the p.Arg516Leufs *51 (c.1546_1548delinsT) variant, found in heterozygosity, is located in exon 11 and causes an arginine to be replaced with leucine, changing the reading frame and consequently creating a premature stop codon. Based on the ACMG criteria, this variant was classified as pathogenic. No second pathogenic variant of the *CFTR* gene was identified in the patient who had this variant. However, 1 to 2% of patients with a well-established clinical diagnosis of CF do not receive a molecular diagnosis even after an extensive, reliable analysis of exonic and flanking regions. One of the main reasons may be the presence of unknown deep intronic variants and variants in regulatory regions [26, 27].

The variant of uncertain significance p.Lys162Glu (c.484 A > G) is located in the transmembrane domain MSD1 (membrane-spanning domain), which lines the channel and, if modified, disrupts lateral permeation of chloride [28]. Several in silico predictors (CADD, Mutation Taster, Polyphen-2) suggest that this substitution is potentially deleterious and indicate that this region is highly conserved across species. There are also two variants in neighboring codons already associated with CF (p.Leu159Ser and p.Tyr161Asp), suggesting that this region is important for the proper functioning of the CFTR protein [29].

The patients with the p.Lys162Glu variant (c.484 A > G) are monozygotic twins. A segregation study revealed that the p.Lys162Glu variant was inherited from their mother, while the p.Ser549Arg variant is present in their father—that is, the variants are found in *trans* (i.e., in distinct alleles). According to the ACMG criteria, the p.Lys162Glu variant (c.484 A > G) may be classified as probably pathogenic, using criteria PM2 + PM5 + PP3 + PP4. However, it is recommended that a functional study be conducted to better understand this variant.

The variant p.Val1022_Ile1023del (c.3063_3068delAGTGAT), never described before in Brazil, was found in two patients without a family relationship. This variant, previously reported in the literature as c.3067_3072delATAGTG [30], is an in-frame deletion that deletes the amino acids valine and isoleucine at codons 1022 and 1023, respectively, and is located at exon 17. According to recent functional studies, this variant causes defects in traffic and conductance, suggesting that it can be classified as a class II variant. These studies also suggest partial functional rescue can be achieved using triple therapy with Elexacaftor, Tezacaftor, and Ivacaftor [31].

So far, six pathogenic deep intronic variants have been described for the *CFTR* gene [32]. One of them, c.2989–313 A > T, was identified here in association with the p.Ala209 = variant. Recent studies using in silico prediction tools suggest that the intronic variant promotes the activation of a cryptic splicing donor site that creates a 118-base-pair pseudoexon and a premature stop codon and possibly leads to the production of a truncated protein [32]. The c.2989–313 A > T variant is also known to be located in a region of high homology with other regions of the genome. In at least four patients in this study from three different families, this variant was found in *cis* (i.e., in the same allele) with the synonymous variant p.Ala209=, constituting a c.[627 A > G; 2989–313 A > T] haplotype. Bergougnoux et al. (2019) recommends that an active search for variant c.2989–313 A > T should be performed when only the p.Ala209 = variant is identified in a patient. In our case 9, a segregation study was carried out on the parents, and it was confirmed that the haplotype was found in *trans* with the p.Phe508del variant.

The p.Thr1299Ile (c.3896 C > T) variant, located in nucleotide-binding domain 2, also known as NBD2, was identified in our case 2. This domain is one of those responsible for ATP binding and hydrolysis, providing energy for closing the channel [28, 33]. Several in silico predictors (REVEL, MutPred, CADD, Mutation Taster, SIFT, Polyphen-2) suggest that this substitution is potentially deleterious and that this is a well-conserved domain across species. This variant is not recorded in the CFTR2 database and is classified here as a variant of uncertain significance due to the scarcity of clinical information available and the lack of functional studies on it [34].

The variant c.-9_14del23, described in our case 8, is absent in population controls and causes the loss of the start codon (p.?), probably preventing protein translation. For this reason, this variant was considered pathogenic. This rare variant was previously known from six patients; the present study increases this number to seven [35].

These findings demonstrate how challenging molecular diagnosis of CF can be, mainly due to a high heterogeneity of variants and genotypes and the difficulties involved in predicting variants’ pathogenicity [36]. Furthermore, in highly multiracial countries such as Brazil, establishing screening panels restricted to the most frequent variants in the country can lead to the underdiagnosis of disease-causing variants [4]. As an example, if the molecular investigation we performed in Sergipe had only used screening panels based on variants with a frequency higher than 1% in individuals with CF in the Brazilian population as a whole, we would have failed to identify approximately 27% of the variants in the patients included in our study—especially the p.Ser549Arg variant. This variant, present in 0.69% of CF patients in Brazil and 0.065% worldwide, was found in 12% (7/58) of our patients from Sergipe. It makes them eligible for treatment with the only modulator drug the Brazilian public health system provides, Ivacaftor. Approximately 82.7% of the patients included in this study had variants that make them eligible for some type of CFTR modulator therapy.

The use of CFTR Next Generation Sequencing revealed rare and previously undescribed pathogenic variants. It will make it possible to estimate the cost of modulator therapy and implement it in the coming years.

## Conclusions

Malnutrition and electrolyte imbalances were the most frequent phenotypes for ages < 2 years, associated with genotypes with two class I–III variants, while bronchiectasis and cases of pneumonia occurred predominantly in patients aged > 10 years and were not related to specific genotypes. The p.Gly542, p.Arg334Trp, and p.Ser549Arg alleles figured among the most frequent variants in this population, and rare and previously undescribed variants were detected among the patients studied.

## Data Availability

All data generated or analysed during this study are included in this published article.

## Abbreviations

ACMG: American College of Medical Genetics
BMI/A: BMI/age
BMI: Body Mass Index
CF: Cystic Fibrosis
CFTR: Cystic Fibrosis Transmembrane Conductance Regulator
EI: Electrolyte imbalances
FEV1: Forced expiratory volume in 1 s
FVC: Forced vital capacity
G1: Group 1
G2: Group 2
G3: Group 3
H/A: Height/age
HAZ: H/A z-score
HGVS: Human Genome Variation Society
IRT: Immunoreactive trypsinogen
MSD1: Membrane-spanning domain
NBS: Newborn screening
NGS: Next-generation sequencing
W/A: Weight/age
WAZ: W/A z-score
